# Preparation of Monolayer Photonic Crystals from Ag Nanobulge-Deposited SiO_2_ Particles as Substrates for Reproducible SERS Assay of Trace Thiol Pesticide

**DOI:** 10.3390/nano10061205

**Published:** 2020-06-19

**Authors:** Changbo Zhang, Jiying Xu, Yi Chen

**Affiliations:** 1 Key Laboratory of Analytical Chemistry for Living Biosystems, Institute of Chemistry, Chinese Academy of Sciences, Beijing 100190, China; zhangchangbo@iccas.ac.cn (C.Z.); xujy@iccas.ac.cn (J.X.); 2 University of Chinese Academy of Sciences, Beijing 100049, China; 3 Beijing National Laboratory for Molecular Sciences, Beijing 100190, China; 4 Huaiyin Institute of Technology, Huaian 223001, China

**Keywords:** monolayer photonic crystal, silica particles, silver nanobulge deposition, SERS assay, pesticide determination

## Abstract

Surface-enhanced Raman scattering (SERS) greatly increases the detection sensitivity of Raman scattering. However, its real applications are often degraded due to the unrepeatable preparation of SERS substrates. Herein presented is a very facile and cost-effective method to reproducibly produce a novel type of SERS substrate, a monolayer photonic crystal (PC). With a building block of laboratory-prepared monodisperse SiO_2_ particles deposited with space-tunable silver nanobulges (SiO_2_@nAg), a PC substrate was first assembled at the air–water interface through needle tip flowing, then transferred onto a silicon slide by a pulling technique. The transferred monolayer PCs were characterized by SEM and AFM to have a hexagonal close-packed lattice. They could increase Raman scattering intensity by up to 2.2 × 10^7^-fold, as tested with p-aminothiophenol. The relative standard deviations were all below 5% among different substrates or among different locations on the same substrate. The excellent reproducibility was ascribed to the highly ordered structure of PCs, while the very high sensitivity was attributed to the strong hotspot effect caused by the appropriately high density of nanobulges deposited on SiO_2_ particles and by a closed lattice. The PC substrates were validated to be applicable to the SERS assay of trace thiol pesticides. Thiram pesticide is an example determined in apple juice samples at a concentration 10^2^-fold lower than the food safety standard of China. This method is extendable to the analysis of other Raman-active thiol chemicals in different samples, and the substrate preparation approach can be modified for the fabrication of more PC substrates from other metallic nanobulge-deposited particles rather than silica only.

## 1. Introduction

Raman spectroscopy has been extensively utilized in the identification and characterization of various chemical and/or biological compounds based on their vibrational fingerprints [1]. It is also, in nature, a fast and label-free analytical tool suitable for quantification, but its primitive methods are very insensitive. Many sensitivity-enhanced strategies [2] have since been explored, of which metallic-based surface-enhanced Raman scattering (SERS) is able to increase the sensitivity by more than a million times. After the cooperation of a surface plasmon polarization (SPP) mechanism with a hotspot effect existing between the metallic nanogaps [3], SERS can increase the sensitivity up to 10^8^-fold in the best case, enabling the detection of even a single molecule [3,4]. It could be also applicable to the analysis of foods [4,5,6,7,8], environmental pollutants [4], and various biological samples in life sciences [2,3,9]. However, in these practical applications, SERS easily encounters the issues of unrepeatable and/or irreproducible measurements. This instability lies majorly in the irreproducible preparation of SERS substrates. As known, a published method may hardly be copied in the case of using metal nanoparticle clusters, which are normally prepared by random aggregation growth, and their uniformity is hardly regulated. Recently, self-assembly and top-down lithography [10,11,12] techniques have been explored for 2D and 3D ordered substrates [10,11,12,13]. Unfortunately, lithographic fabrication is often operated under very costly conditions. Practically, assembling approaches are a better choice to lower the cost. Xiong et al. demonstrated that an evaporation-induced self-assembly strategy was able to vertically align a monolayer of Au nanorods with nominal gaps at 0.8 nm [14]. Such an assembly can be mediated by DNA, block copolymers and molecular ligands [15,16,17,18], which helps to produce a stronger hotspot effect. The present assembling methods have several disadvantages. Firstly, the fabrication is complicated due to the ligands needed to stabilize the nanoparticles. Secondly, the assembling approaches still lack fabrication reproducibility [19].

**Scheme 1:**
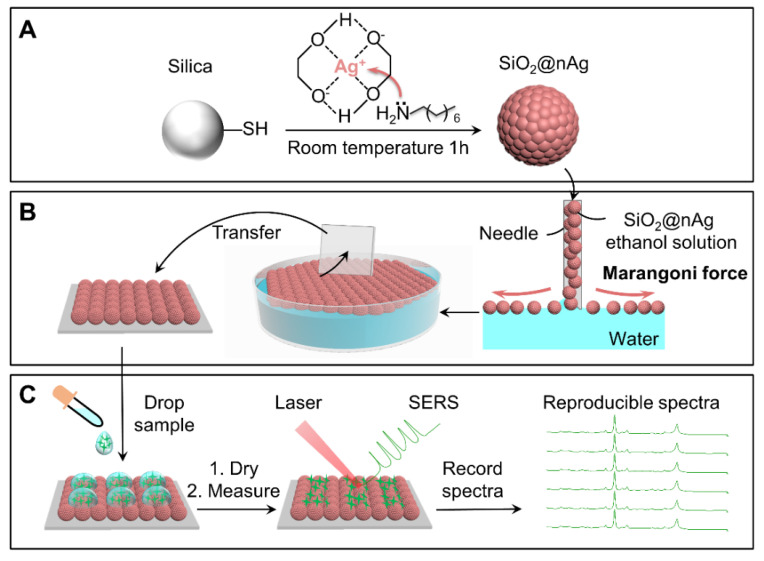
Schematic illustration of (**A**) the fabrication process for monodisperse and space-tunable SiO_2_@nAg, and (**B**) assembly of a monolayer photonic crystal (PC) SERS substrate from silver nanobulge-deposited silica particles (SiO_2_@nAg) on a silicon slide for (**C**) SERS detection of an analyte after the drying of its droplet.

We have therefore tried to develop a ligand-free, more reproducible assembling method, inspired by our works on the fabrication of photonic crystals (PCs) from particles [20,21,22,23]. Herein presented is an innovative method to produce monolayer PCs on silicon slides for an SERS assay of thiol substances. The monolayer PC substrates were self-assembled from silica particles deposited with space-tunable silver nanobulges (SiO_2_@nAg). They were first assembled on the water surface by a needle tip flow (NTF) technique [24,25,26], then transferred onto silicon slides by pulling the slides from beneath the particle layer (Scheme 1). The on-water assembly was modified from a reported method for the assembly of a monolayer film from hydrophilic metal nanoparticles at the toluene–water interface [27], where the toxic toluene [28] was replaced by air on a set of laboratory-fabricated devices. The novel PC substrates could thus be fabricated reproducibly, greenly and cost-effectively, and in turn used to largely improve the sensitivity and reproducibility of SERS determination.

## 2. Materials and Methods 

### 2.1. Materials and Reagents

Ammonium hydroxide (28.0–30.0 wt%, NH_3_), tetraethylorthosilicate (TEOS, 98.0%), and 3-mercaptopropyl trimethoxysilane (MPTS, 95.0%) were purchased from Alfa Aesar (Shanghai, China). Ethylene glycol (EG, 99.0%) was from Aladdin (Shanghai, China). Polyvinylpyrrolidone (PVP, Mw = 40,000) and octylamine (OA, 99.5%) were from Sigma-Aldrich (St. Louis, MO, USA). Anhydrous ethanol (99.5%) was from Beijing Chemical Works (Beijing, China) and silver nitrate (AgNO_3_, 99.8%) from Guanghua Sci-Tech Co., Ltd. (Guangzhou, China). Thiram was purchased from J&K scientific (Beijing, China). All chemicals and reagents were used as received without further purification. Deionized (DI) water was purified by a Milli-Q water filtration system (Millipore, Milford, MA, USA). Apple juice (Huiyuan) was purchased from a local supermarket in Beijing.

### 2.2. Synthesis of SiO_2_@nAg

Monodisperse silica spheres were synthesized according to our previous work, modified from the Stöber method [23], and dispersed at 50.0 mg/mL in ethanol. The silica surface was further modified to have a thiol terminal [29] as follows: in a single-port glass flask, the silica dispersion (6.0 mL at 50.0 mg/mL, equal to 300 mg silica in total), 250 µL MPTS and 50.0 µL ammonium hydroxide (28.0−30.0 wt%) were added in sequence and mixed by stirring at 25 °C for 10 h, and the resulted spheres were cleaned with ethanol more than 5 times. A small quantity (1.00–30.0 mg) of the resulting SH-functionalized silica spheres was mixed first with 5.00 mg of PVP in 25 mL EG, then with 30.0 mg AgNO_3_ in 25 mL EG. After 41.5 µL OA was quickly added, the mixture could react at 25 °C for 1 h. The resulting SiO_2_@nAg particles were harvested by centrifugation and cleaned with ethanol at least 3 times.

### 2.3. Preparation of SiO_2_@nAg-Based PC and Other Substrates

The process to fabricate SiO_2_@nAg-based PC substrate on silicon slides by the NTF technique is illustrated in Scheme 1B. An ethanol suspension of 2.0 mg/mL SiO_2_@nAg was vertically delivered onto an air–water surface in a round glass petri dish (6 cm diameter × 3 cm depth). The flow rate was adjusted by a peristaltic pump, via a 5-mL plastic syringe with a 21-gauge needle. When the needle tip rightly contact the water surface, a meniscus structure forms along the needle tip wall, and the particles spread out along the surface according to surface tension gradients and/or Marangoni force until all the surface is occupied. The flow was then stopped to avoid the particles dispersing into the bulk water. A silicon slide (4 mm × 4 mm) was carefully inserted into the water beneath the SiO_2_@nAg monolayer and then lifted off the particle layer. The slide was ready to use after drying at room temperature.

The non-PC substrates were prepared either by in-situ evaporation of droplets of monodisperse SiO_2_@nAg suspension on silicon slides or self-assembly of polydisperse SiO_2_@nAg particles on the water surface through the above-described NTF technique.

### 2.4. PC-Based SERS Assay of Thiram in Food 

A stock solution of thiram (100 ppm) was prepared by dissolution of 5.00 mg thiram in 50.0 mL ethanol. A series of thiram standard solutions (0.00, 50.0, 100, 200, 400, 600, 800, 1.00 × 10^3^ ppb) were prepared by dilution of the stock solution with ethanol. Testing samples (0.00, 50.0, 100, 200, 400, 600, 800, 1.00 × 10^3^ ppb) were prepared by spiking an appropriate volume of the stock solution into a 5.00 mL commercial apple juice and diluting with water to 50.0 mL without any pretreatment. The process for SERS detection on the monolayer SiO_2_@nAg PC substrate is illustrated in Scheme 1C. Specifically, an aliquot of 10.0 µL testing or standard sample solution was directly spotted on a monolayer SiO_2_@nAg PC substrate and in-situ-dried at room temperature. The Raman spectra were then measured by use of a portable i-Raman@plus 785S spectrometer (B&W TEK, Newark, DE, USA), equipped with a high-quantum-efficiency charge-coupled device (CCD) array detector in backscattered configuration, coupled with a BAC151B Video Microscope and a 20x objective (105 µm laser spot size, N.A. = 0.4). All Raman spectra were frequency calibrated with silicon peaks before each spectrum acquisition. The Raman spectra were acquired under a 785-nm diode laser at a power of 4.0 mW, with a 6-s exposure and one-time accumulation. All the reported Raman spectra were background-corrected by means of an asymmetric least-square smoothing technique (asymmetric factor 0.001, threshold 0.05, smoothing factor 4, number of iterations 10).

### 2.5. Other Measurements

The extinction spectra of SiO_2_@nAg were measured on a UV spectrometer, model UV-1800, from Shimadzu (Kyoto, Japan). The morphology and size of SiO_2_@nAg were characterized using a scanning electron microscope of model SEM-4800 and a transmission electron microscope of model TEM-1011, both from JEOL (Tokyo, Japan). The morphology of the monolayer SiO_2_@nAg PC was also measured using an atomic force microscope of model AFM-Dimension Edge from Bruker (Billerica, MA, USA).

The diameter of the SiO_2_@nAg particles and the Ag nanobulge interval were measured using image analysis by Nano Measure software. At least 150 randomly selected SiO_2_@nAg particles in certain TEM images were averaged to reduce random errors. The Ag nanobulge size was calculated by subtracting the diameter of the SiO_2_ particle from the diameter of the SiO_2_@nAg particle.

## 3. Results and Discussion

### 3.1. Conditions to Synthesize SiO_2_@nAg

**Figure 1:**
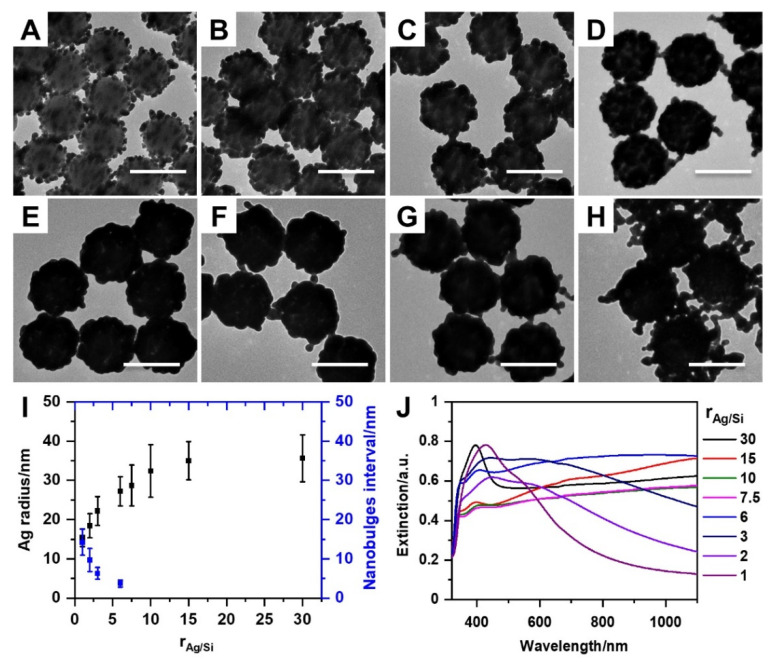
TEM images of SiO_2_@nAg, obtained at a weight ratio of AgNO_3_ to SiO_2_ (r_Ag/Si_) of (**A**) 1, (**B**) 2, (**C**) 3, (**D**) 6, (**E**) 7.5, (**F**) 10, (**G**) 15 and (**H**) 30, with (**I**) an extracted size of Ag nanobulges and (**J**) corresponding extinction spectra. All scale bars in Figure (**A**–**H**) correspond to 200 nm.

In order to reproducibly assemble PC-based SERS substrates, monodisperse and space-tunable SiO_2_@nAg particles have to be available. A seedless, one-step deposition was developed to synthesize the required particles at room temperature over 1 h. This mild synthetic condition results from the formation of an Ag•EG complex, which helps the reduction of Ag^+^ to Ag [29,30]. To ensure reproducible preparation, three key factors should be well controlled. Firstly, the kernel silica spheres should have a polydispersity index (σ/d) < 2% [31], and the monodisperse silica we used could be easily produced by the separation-coupled assembly method [23]. Secondly, uniform Ag nanobulges should be firmly deposited on the silica spherical surface, which was achieved by reaction with MPTS to obtain a thiol terminal with a high affinity to Ag. Without this terminal, Ag nanoparticles will form away from or near the silica spheres rather than on their surface (see Figure S1). Thirdly, the size of Ag nanobulges and their interval on the silica spheres should be well regulated to provide sufficient hotspots, which was realized by adjusting the weight ratio of AgNO_3_ to silica (r_Ag/Si_) in a pre-synthesis solution (Figure 1A–I). It was found that SiO_2_@nAg-6 particles prepared at r_Ag/Si_ = 6 gave the best SERS signals, measured by varying the silica weight while fixing AgNO_3_ at 30.0 mg. Figure 1I illustrates the size variation in the Ag nanobulges and Figure 1J the corresponding extinction spectra caused by SPP and LSPP. The SiO_2_@nAg-6 particles with 27 ± 4 nm Ag nanobulges at an average interval of 3 ± 1 nm offered almost the highest SERS signals (Table S1). At r_Ag/Si_ below 6, the nanobulges decreased their size as r_Ag/Si_, down to 15 ± 2 nm at r_Ag/Si_ = 1, with an increase in their interval up to 14 ± 3 nm. This was accompanied by reducing of the hotspot effect and in turn of SERS signals. Oppositely, at r_Ag/Si_ above 7.5 (Figure 1E–H), the nanobulges became too crowded to provide sufficient space for the hotspot effect. In fact, the nanobulges fused into a 30-nm-thick shell, which was further thickened up to 40 nm at r_Ag/Si_ = 30 (Figure 1H). This produced more and more uneven SiO_2_@nAg particles due to the random growth of Ag nanoparticles on the shell. It should be noted that r_Ag/Si_ was found to depend a bit on the diameter of the kernel spheres, or more exactly, r_Ag/Si_ slightly increased with the surface curvature of the kernel spheres, varying between 5 and 7 with the diameter of the kernel silica spheres between 300 nm and tens of nanometers. For example, r_Ag/Si_ approached 5 in the case of 266 nm silica particles.

These changes were also confirmed by the extinction spectra of SiO_2_@nAg (Figure 1J). The SPP peak appeared between 400 nm and 500 nm after the Ag nanobulges were deposited on the silica surface. The SPP peak became broad and flat as r_Ag/Si_ increased, also causing red-shift of the wavelength. At about r_Ag/Si_ = 6, the spectra of the particles changed to broad bands that spread from the visible to the NIR region. Furthermore, a blue-shifted band appeared due to the coupling of SPP between the internal and external surfaces of the Ag shell [30]. The absorption peak between 400 nm and 500 nm appeared once again as Ag nanobulges re-deposited on the Ag shell surface. Nevertheless, this re-deposition should be avoided to preserve Ag and to achieve better reproducibility.

**Figure 2:**
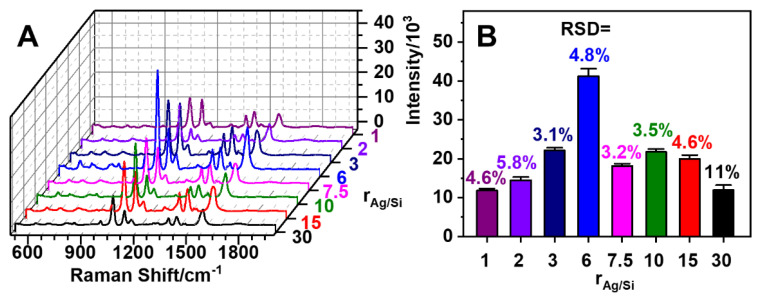
Effect of the weight ratio of AgNO_3_/SiO_2_ on (**A**) Raman signals and (**B**) signal intensity at 1078 cm^−1^ for pATP on SiO_2_@nAg. The Raman spectra were acquired at 1.0 × 10^−4^ M pATP using a 785-nm laser at 4.0 mW output power for 6 s. Each spectrum in Figure A was averaged over 15 random measurements from triplicate experiments.

The final confirmation was performed by SERS of a testing sample, p-aminothiophenol (pATP). Through spotting 10.0 µL of 1.0 × 10^−4^ M pATP solution onto monolayer SiO_2_@nAg PC substrates prepared at different r_Ag/Si_, SERS spectra were recorded by excitation at 785 nm (Figure 2A). The variation trend was the same: the characteristic Raman bands [9] recorded at 1078 cm^−1^ first increased with r_Ag/Si_ and then reduced, with the maximal signal at r_Ag/Si_ = 6 (Figure 2B). Unexpectedly, the SERS signal decreased sharply on SiO_2_@nAg-7.5 even if its shape did not vary too much from that of SiO_2_@nAg-6 (Figure 1D,E). This implies that the hotspot effect happens only at a very narrow gate. The control of r_Ag/Si_ at about 6 is thus suggested to be critical.

In addition to the influence of the weight ratio of AgNO_3_ on silica, we further examined the dependence of SERS performance on the size of the kernel silica spheres. Figure S2A–D shows the TEM images of SiO_2_@nAg prepared with different sizes of silica spheres (130–370 nm). All the obtained SiO_2_@nAg particles possessed a uniform nanostructure and were monodisperse. Figure S3A demonstrates a set of averaged Raman spectra of pATP (1.0 × 10^−6^ M) measured on PC substrates assembled from different sizes of SiO_2_@nAg. Obviously, all the SiO_2_@nAg exhibited excellent SERS abilities, and the signal intensities at 1078 cm^−1^ first increased as the particle size increased and then decreased, with the maximal signal at 266-nm SiO_2_@nAg (Figure S3B). A possible reason is that 266-nm SiO_2_@nAg provided almost the largest size Ag nanobulges at the same interval (Table S2).

### 3.2. Assembly of Monolayer PCs from SiO_2_@nAg

Monolayer SiO_2_@nAg PCs can be fabricated on a large area (>28 cm^2^) in 2 min using the NTF technique (Figure S4A). A key point is to use ethanol as a dispersion solvent to reduce the surface tension during the assembly of SiO_2_@nAg particles on the water surface. With this design, the target particles could spread fast, due to the Marangoni effect, from the delivery needle tip along the water surface, outward until the border (Video S1 in Supplementary Materials). This produces a continuous floating monolayer of particles on the water face. The monolayer can be easily transferred onto a silicon slide by gently lifting the slide from beneath the particle layer.

**Figure 3:**
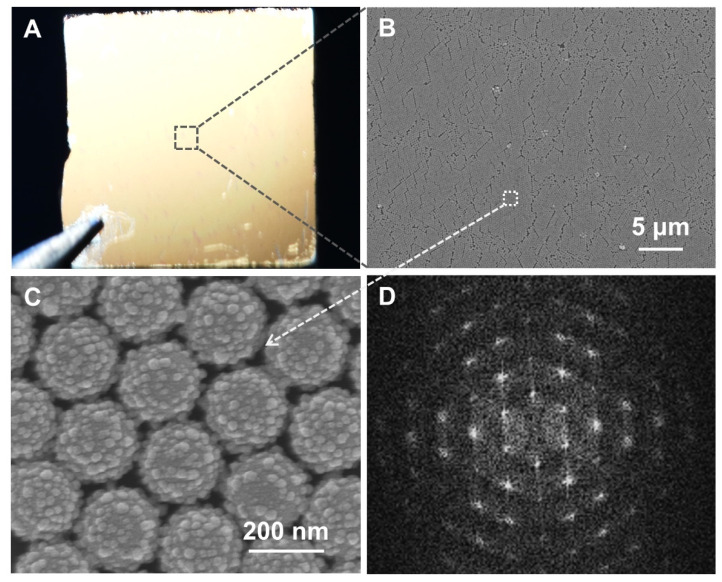
(**A**) Photograph, (**B**,**C**) SEM images, and (**D**) FFT pattern of a SiO_2_@nAg-6-assembled monolayer PC substrate transferred onto a silicon slide. Figure D reveals a hexagonally packed lattice.

Ordering a monolayer of SiO_2_@nAg on the water surface depends very much on the flow rate of the particle suspension. This can be demonstrated by qualitatively viewing the SEM images, as shown in Figure 3 and Figure S5. The transferred monolayer PC of SiO_2_@nAg-6 formed at a ≥0.2 mL/min flow rate is clearly less ordered, with more randomly oriented 2D crystallites (Figure S5), than at a lower flow rate. In other words, the monolayer PCs obtained at a <0.2 mL/min flow rate are better ordered. The transferred monolayer PC of SiO_2_@nAg-6, bright yellow (Figure 3A), was characterized by SEM and the fast Fourier transform (FFT) pattern to have a 2D hexagonal lattice (Figure 3B–D). The “monolayer” of the transferred PCs was confirmed by the height curve extracted from the AFM image (Figure S6), giving an average height of 220 nm, very close to the average diameter of SiO_2_@nAg-6 at 219 nm. Therefore, the flow rate is better kept below 0.2 mL/min. More tests showed that the NTF technique could be used to assemble other monolayer PCs at a required size, not just limited to SiO_2_@nAg-6 (Figure S2E–H). A large size of monolayer PCs facilitates the reproducible performance of SERS on different locations of the same substrate and/or its cut pieces. As demonstrated in Figure S4B,C, the Raman spectra acquired from the three different regions of a same PC substrate are very comparable, with peak height (at 1087 cm^−1^) variation below 5% (4.7% for the central region, 3.9% for the middle ring, and 5.0% for the outer part).

### 3.3. SERS Performance of SiO_2_@nAg-Based Monlayer PCs 

By testing with 1.0 × 10^−8^–5.0 × 10^−5^ M pATP standard solutions, monolayer PCs assembled from 266-nm SiO_2_@nAg were shown to be capable of a large increase in SERS signals depending on analyte concentration (Figure S7). The lowest measurable concentration approached 10 nM pATP at 1078 cm^−1^. This is about seven orders of magnitude lower than 0.1 M pATP for non-SERS measurement. More exactly, the enhancement factor (*EF*) was 2.2 × 10^7^, calculated by following equation:

(1) $$EF=\frac{{Ι}_{SERS}\times N_{ref}}{{Ι}_{ref}{\times N}_{SERS}}$$

where *I_SERS_* is the peak intensity at 1078 cm^−1^ measured from a 1.0 × 10^−6^ M pATP spotted on the PC substrate, while *I_ref_* is the intensity measured at 0.1 M pATP on a silicon reference substrate (Figure S8) and *N_SERS_* and *N_ref_* are the number of pATP molecules excited by the laser beam on the SiO_2_@Ag-based PC and silicon reference substrates, respectively. The peak at 1078 cm^−1^ was chosen as it is the characteristic band of pATP, and the variation in its intensity represents the change in the pATP’s quantity. Considering the intensity of the whole spectrum will lose the focus on pATP. The peak at 1078 cm^−1^ is a more suitable signal for quantitative analysis than other characteristic peaks. Therefore, we only chose the peak at 1078 cm^−1^ to discuss our results. These EFs were comparable with the 3.2 × 10^7^ measured for AAO-template-assisted deposition of Ag nanoparticle substrates [32], and better than the 1.8 × 10^6^ measured on a vertically aligned monolayer of CTAB-stabilized Au nanorods that were prepared by an evaporation-induced self-assembly method [14].

**Figure 4:**
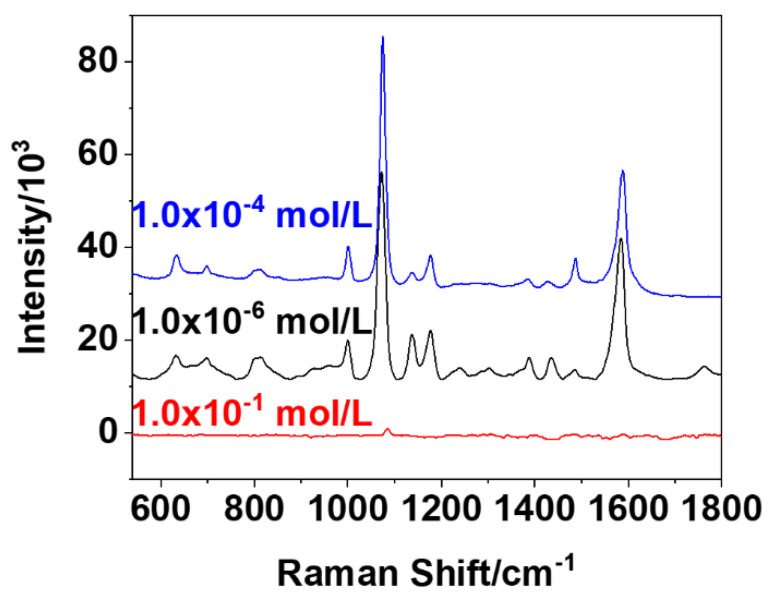
Raman spectra acquired at 0.1 M pATP on a bare silicon surface (**bottom**), at 1.0 × 10^−6^ M pATP on a 266-nm SiO_2_@nAg-assembled monolayer PC substrate (**middle**), and at 1.0 × 10^−4^ M pATP on a silicon slide deposited with isolated 266-nm SiO_2_@nAg particles (**upper**).

**Figure 5:**
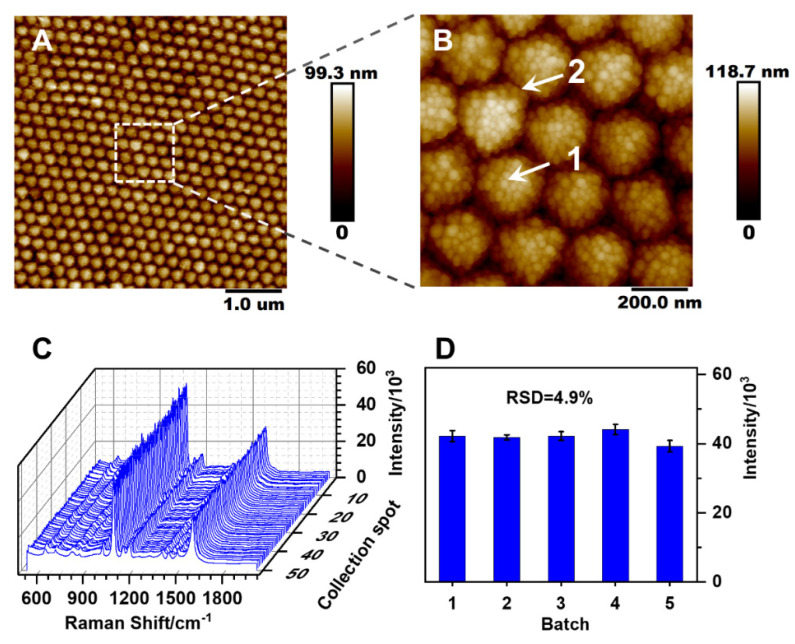
(**A**,**B**) AFM images of a SiO_2_@nAg-assembled monolayer PC substrate on a silicon slide, (**C**) 50 SERS spectra collected from different locations on the same PC substrate, and (**D**) the statistical deviation of SERS intensities of pATP at 1087 cm^−1^ acquired from five different PC substrates assembled in five batches.

Further experiments showed that the EFs measured on 266-nm SiO_2_@nAg particle-island substrates were about two orders of magnitude lower than those on monolayer PCs (Figure 4). This implies that there are two types of structures responsible for the hotspot effect: the first is the gaps between the Ag nanobulges (arrow 1 in Figure 5B), and the second is those between the SiO_2_@nAg particles (arrow 2 in Figure 5B). The first type of hotspot effect is dependent on the size of the Ag nanobulges and their interval, which has been discussed above, while the second type of hotspot effect is determined by the space between the SiO_2_@nAg particles; these should be as close as possible since the very large particles are hard to pack very closely due to their repulse force. This study revealed that the on-water surface assembly of particles is an easy way to pack them tightly, giving a hexagonal close lattice of SiO_2_@nAg particles (Figure 5A,B).

As indicated, the PC substrates could largely avoid the irreproducible SERS on the “same” substrates, which can be further validated by the Raman peak height measured at 1078 cm^−1^: the relative standard deviation (RSD) was below 3.8% (Figure 5C and Figure S8) among 50 measurements on the same substrate and below 4.9% on five different PCs (Figure 5D). This excellent reproducibility should be clearly ascribed to the very ordered structure of the PC substrates, including the extremely uniform kernel silica particles used. The opposite evidence lies in the use of non-PC substrates (Figure S9), obtained by either monodisperse particles (with polydispersity index << 2%, Figure S9A) or polydisperse particles (with polydispersity index > 2%, Figure S9B). Although the non-PC substrates from polydisperse particles gave somewhat better Raman spectra (Figure S9D), with less variation in peak height (Figure S9F), than the substrates of monodisperse particles (Figure S9C,E), the RSDs of peak height were not better than 15% (Figure S9F), or, even worse, down to 35% (Figure S9E).

### 3.4. Methodological Features and Application

**Figure 6:**
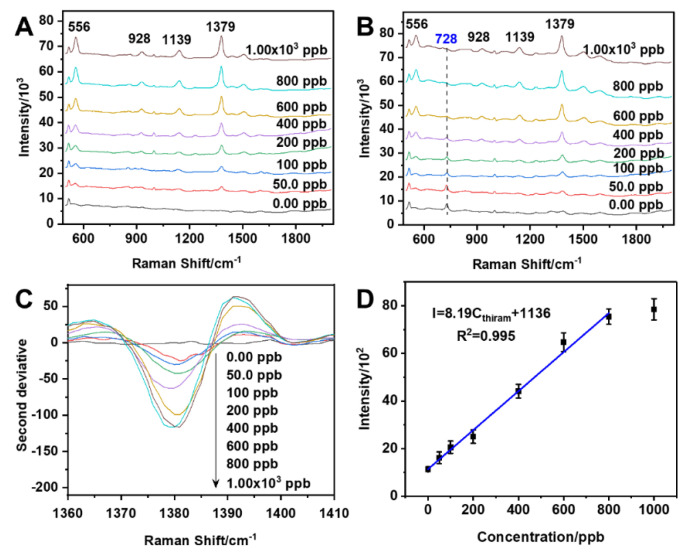
Averaged Raman spectra of 0.00~1.00 × 10^3^ ppb thiram in (**A**) ethanol and (**B**) spiked in a diluted apple juice sample; (**C**) the second derivatives of the spectra A at 1379 cm^−1^ and (**D**) linear curve plotted based on the spectra B at 1379 cm^−1^. The spectra in Figure **A**,**B** were averaged over 15 random measurements from triplicate experiments. The spectra at 0 ppb suggest that background signals do not affect SERS measurement.

The PC-based SERS method was validated to be applicable to the determination of thiram in apple juice samples. Thiram is a dithiocarbamate often used as a seed dressing to prevent fungal diseases, and/or as preservative to preserve mature fruits and vegetables during storage [6,8,29,33]. In apple groves, it is used to avoid the melanosis or canker of apples [8,33]. However, improper and/or overuse of thiram pesticides increases human health risk and environmental pollution stress. It must be well regulated in respect of the quantity applied. Herein, we show that SiO_2_@nAg-assembled monolayer PC substrates are suitable for direct SERS determination of thiram in apple juice samples. The measurement was performed by dotting sample solution(s) on a monolayer PC substrate, normally without any sample pretreatments. Raman spectra were recorded after the drying of the spots, as illustrated in Figure 6, where the strongest peak at 1379 cm^−1^ (symmetric deformation of CH_3_ weakly coupling to C-N stretching) [8,29] is the most suitable signal for quantitative analysis over the other characteristic peaks at 556 (S-S stretching), 928 (CH_3_-N stretching) and 1139 (CH_3_ rocking weakly coupling to C-N stretching). The exact wave number at 1379 cm^−1^ was fixed by the second derivatives (Figure 6C), which help to reduce the disturbance of baseline drifting and enhance the position resolution. The plot (Figure 6D) at 1379 cm^−1^ has a linear range between 50.0 ppb and 800 ppb thiram, with *R*^2^ = 0.995. The limit of detection (S/N = 3) reached 34.7 ppb thiram. Without any sample pretreatments, the SERS assay offered better sensitivity than Ag NS-based SERS [29] and even SPE-UV absorption [34], close to LC-MS [35,36] and ELISA [37] (Table 1). Although less sensitive than the electrochemical method [38], it is two orders of magnitude lower than the general default maximum residue limitation (MRL), 5 mg/L in apple juice, which is regulated by the national food safety standards of China (GB2763-2019). Even in this very sensitive case, there was no thiram determined in apple juices from Beijing local markets (e.g., Huiyuan Apple Juice).

The abundant carbohydrates in the apple juices were shown to display negligible interference with the measurements. Figure 6B illustrates that the averaged Raman spectra of thiram-spiked apple juice samples are nearly the same as the spectra from thiram standards (Figure 6A), except for the peak at 728 cm^−1^, which is reduced as thiram increases. This has no impact on the determination at the peak at 1379 cm^−1^. This selectivity results from the selective formation of Ag-S bonds on the substrate. Other thiol-free substances, such as carbohydrates, are unable to reach the Ag surface closely and in turn become undetectable by Raman scattering for non-hotspot effects (Figure S10). It should be noted that the Raman intensity of thiram was slightly reduced in the real apple juices (Figure 6B) compared with the thiram standards (Figure 6A).

## 4. Conclusions

In conclusion, a novel approach has been established for the successful assembly of monolayer PC substrates from SiO_2_@nAg particles, which were synthesized by reduction of Ag on monodisperse SiO_2_ kernel particles prepared through separation-coupled assembly. This preparation is facile and reproducible, and the resulting monolayer PC substrates make it possible to very stably measure Raman scattering signals suitable for quantification of trace thiol substances. The PC-based assay was able to enhance the detection sensitivity 2.2 × 10^7^-fold, with the relative standard deviation of peak height < 5%. This PC-based SERS assay was validated to be applicable to the determination of trace thiram pesticide in apple juice samples, pushing the limit of detection down to 34.7 ppb. This PC-based SERS assay is expandable to the analysis of other thiol substances than thiram, and the assembling approach is extendable to the preparation of more PC substrates by the replacement of Ag with other metal(s) and/or the kernel particles of silica with other materials.

## Figures and Tables

**Table 1:** Sensitivity comparison of a monolayer PC substrate-based SERS assay of thiram pesticides with other methods.

Detect Method	Limit of Detection	Ref
Electrochemical method	1.61 ppb	[38]
ELISA	40.0 ppb	[37]
LC-MS/MS	25.0 ppb	[35,36]
SPE-UV	330 ppb	[34]
Ag NS-based SERS	14.4 ppm	[29]
Monolayer PC-based SERS	34.7 ppb	this work

## Supplementary Materials

The following are available online at https://www.mdpi.com/2079-4991/10/6/1205/s1, Figure S1: TEM images of Ag nanoparticles formed after mixing with silica spheres without SH terminals; Figure S2: TEM images of SiO_2_@nAg synthesized with different sizes of kernel silica spheres—(A) 136 nm, (B) 210 nm, (C) 266 nm and (D) 375 nm—and AFM images of their corresponding assembled monolayer SiO_2_@nAg PC substrates (E–H), respectively; Figure S3: Effect of the size of silica spheres on (A) Raman signals and (B) signal intensity at 1078 cm^−1^ for pATP on SiO_2_@nAg. The Raman spectra were acquired at 1.0 × 10^−6^ M pATP using a 785-nm laser at a 4.0 mW output power for 6 s; Figure S4: (A) Photograph of a SiO_2_@nAg-6-based monolayer PC on the water surface, (B) Raman spectra of pATP and (C) the average intensity at 1087 cm^−1^ among different locations along the radial direction; Figure S5: SEM image of a SiO_2_@nAg-6 monolayer PC substrate assembled by needle tip flow (NTF) at a particle suspend flow rate >0.2 mL/min; Figure S6: AFM image (A) and height profile (B) of a SiO_2_@nAg-6-based monolayer PC substrate on a silicon slide; Figure S7: Concentration-dependent Raman spectra of pATP spotted on a 266-nm SiO_2_@nAg-assembled monolayer PC substrate excited with a 785-nm laser at 4.0 mW power for 6 s; Figure S8: The variation in the Raman peak height of pATP at 1087 cm^−1^ measured among 50 different locations on the same PC substrate; Figure S9: (A,B) SEM images of non-PC substrates from SiO_2_@nAg with a polydispersity index at (A) << 2% or (B) > 2%, used for (C,D) SERS of pATP to show (E,F) the variations in the peak intensity at 1087 cm^−1^ among 50 measurements, respectively; Figure S10: Raman spectra of different chemicals with or without thiol groups added in a diluted apple juice sample; Table S1: Parameters for the synthesis of Ag nanobulges-deposited silica spheres (SiO_2_@nAg) with data extracted from TEM images; Table S2: Parameters for SiO_2_@nAg of different sizes with data extracted from TEM images; evaluation of enhancement factor; evaluation of the limit of detection; Video S1: The process of assembly of a SiO_2_@nAg monolayer PC on the water surface.
