# Supplementary material for: Preparation of Monolayer Photonic Crystals from Ag Nanobulge-Deposited SiO2 Particles as Substrates for Reproducible SERS Assay of Trace Thiol Pesticide
Source: Nanomaterials (Basel). 2020 Jun 19;10(6):1205. doi: 10.3390/nano10061205 (PMC7353115; doi:10.3390/nano10061205)
Supplement: Supplementary file 1 [file nanomaterials-10-01205-s001.zip › sup-proof-conversion/nanomaterials-835016-supplementary-conversion.pdf]

Supplementary Materials:

Preparation of Monolayer Photonic Crystals from Ag Nanobulge-Deposited SiO_2 Particles as Substrates for Reproducible SERS Assay of Trace Thiol Pesticide

Changbo Zhang [1,2], Jiying Xu [1,2] and Yi Chen [1,2,3,4,*]

[1] Key Laboratory of Analytical Chemistry for Living Biosystems, Institute of Chemistry, Chinese Academy of Sciences, Beijing 100190, China; zhangchangbo@iccas.ac.cn (C.Z.); xujy@iccas.ac.cn (J.X.)
[2] University of Chinese Academy of Sciences, Beijing 100049, China
[3] Beijing National Laboratory for Molecular Sciences, Beijing 100190, China
[4] Huaiyin Institute of Technology, Huaian 223001, China
* Correspondence: chenyi@iccas.ac.cn; Tel.: +86-10-62618240; Fax: +86-10-62559373

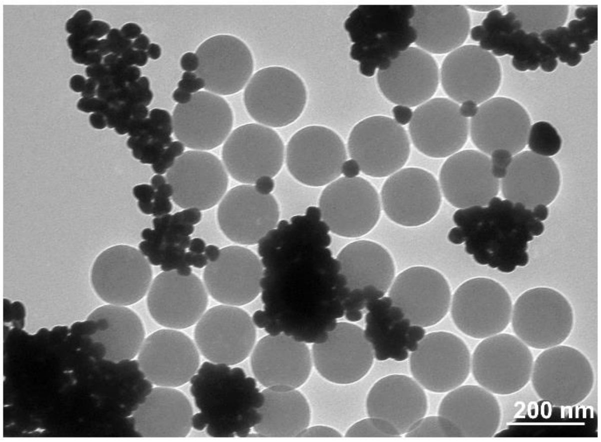

Figure S1. TEM images of Ag nanoparticles formed after mixed with silica spheres without SH-terminal.

Table S1. Parameters for the synthesis of Ag nanobulges-deposited silica spheres (SiO_2@nAg) with data extracted from TEM images.

$r_{Ag/Si}$	Ag nanobulges diamter/nm	Nanobulges interval/nm
1	15±2	14±3
2	18±3	10±3
3	22±4	6±2
6	27±4	3±1
7.5	28±5*	NS
10	32±6*	NS
15	35±5*	NS
30	36±6*	NS

* corresponds to the silver shell thickness of SiO_2@nAg.
NS = no statistics as formation of a complete shell.

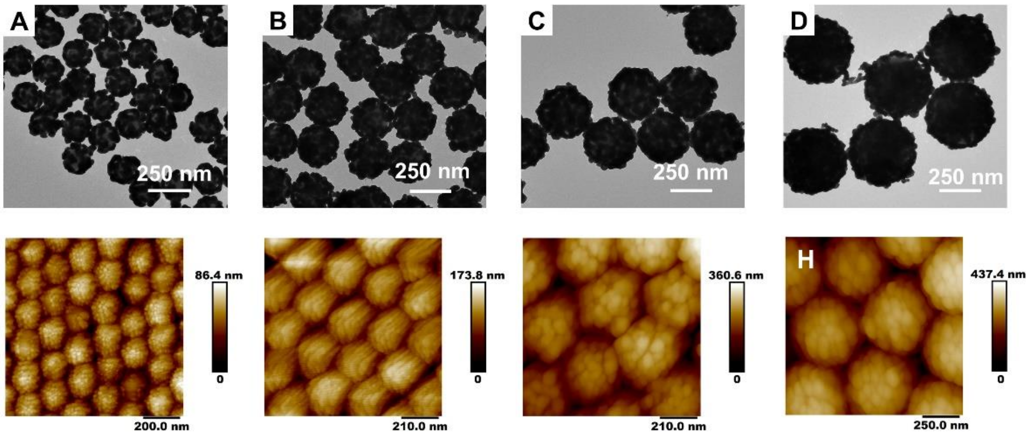

Figure S2. TEM images of SiO_2@nAg synthesized with different size of the kernel silica spheres: (**A**) 136 nm, (**B**) 200 nm, (**C**) 266 nm and (**D**) 367 nm and AFM images of their corresponding assembled monolayer SiO_2@nAg PC substrates (**E**), (**F**), (**G**), and (**H**), respectively.

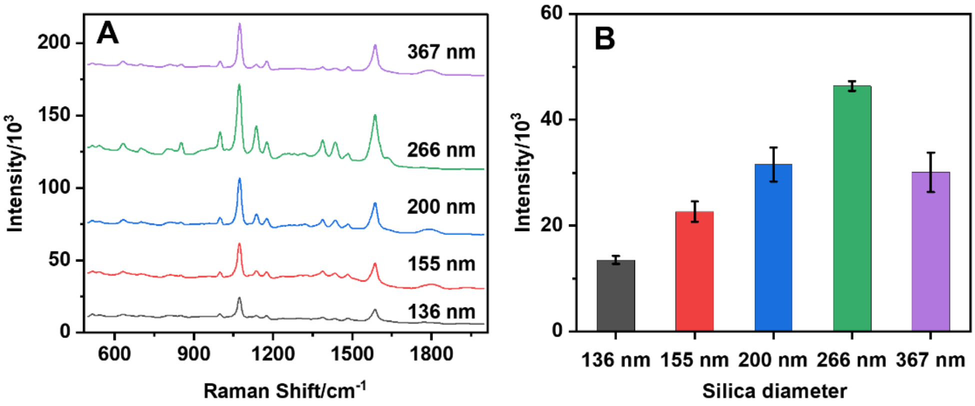

Figure S3. Effect of the size of kernel silica spheres on (**A**) Raman signals and (**B**) signal intensity at 1078 cm^{-1} for pATP on SiO_2@nAg. The Raman spectra were acquired at $1.0x10^{-6}$ M pATP using a 785 nm laser at 4.0 mW output power for 6 s.

Table S2. Parameters for SiO_2@nAg of different size with data extracted from TEM images.

Sample	Ag nanobulges diameter/nm	Nanobulges interval/nm
136 nm-SiO_2@nAg	20±3	5±2
155 nm-SiO_2@nAg	27±4	3±1
200 nm-SiO_2@nAg	30±5	3±1
266 nm-SiO_2@nAg	37±4	3±1
367 nm-SiO_2@nAg	33±6	3±1

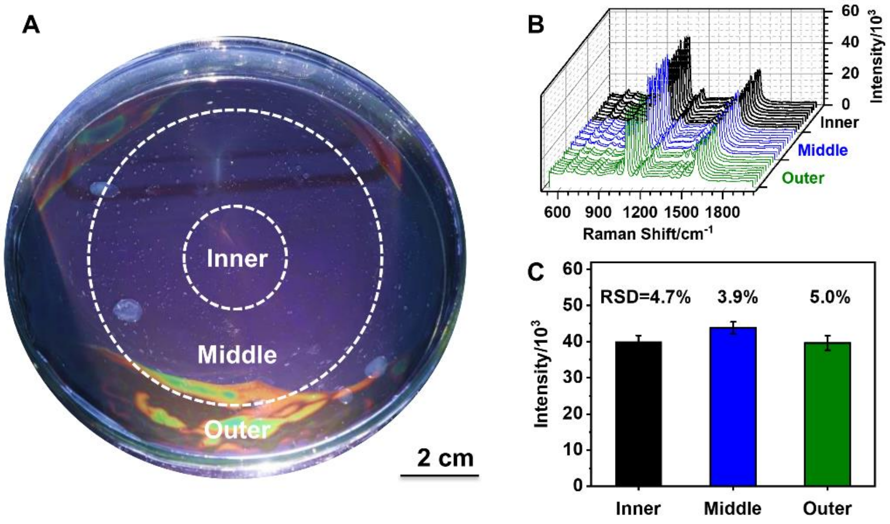

Figure S4. (**A**) Photograph of a SiO_2@nAg-6-based monolayer PC on the water surface, (**B**) Raman spectra of pATP and (**C**) the averaged intensity at 1087 cm^{-1} among different locations along the radial direction.

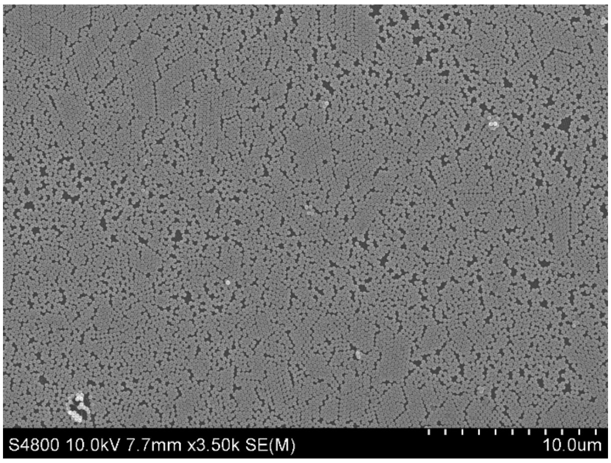

Figure S5. SEM image of SiO_2@nAg-6 monolayer PC substrate assembled by needle tip flow (NTF) at a particle suspension flow rate > 0.2 mL/min.

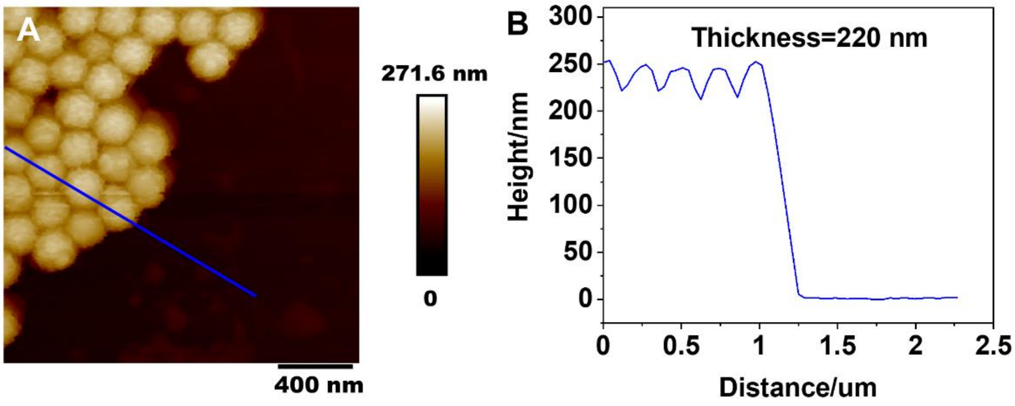

Figure S6. AFM image (**A**) and height profile (**B**) of SiO_2@nAg-6-based monolayer PC substrate on a silicon slide.

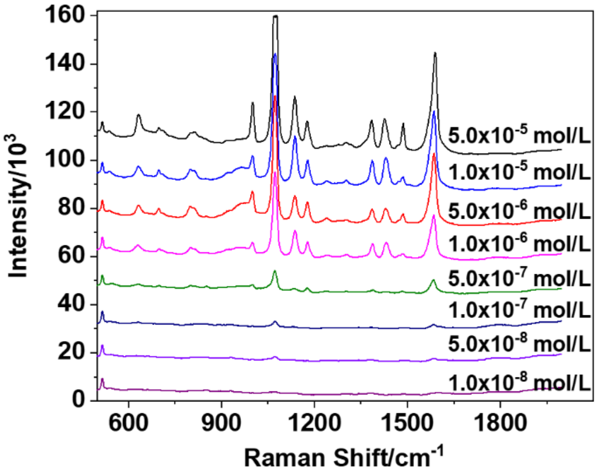

Figure S7. Concentration-dependent Raman spectra of pATP spotted on a 266 nm-SiO_2@nAg-assembled monolayer PC substrate excited at 785 nm laser and 4.0 mW power for 6 s.

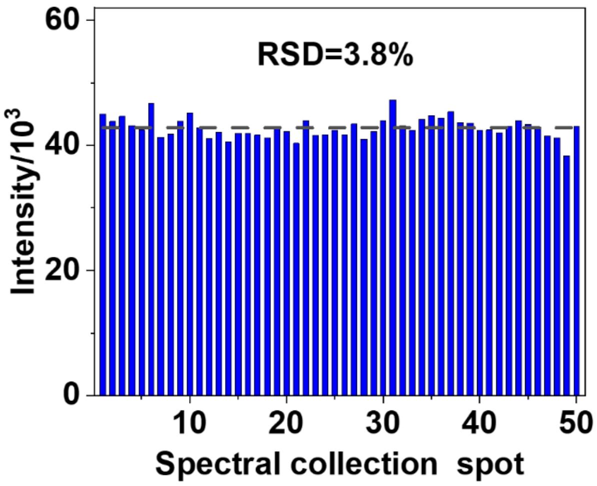

Figure S8. The variation of the Raman peak height of pATP at 1087 cm^{-1} measured among 50 different locations on a same PC substrate.

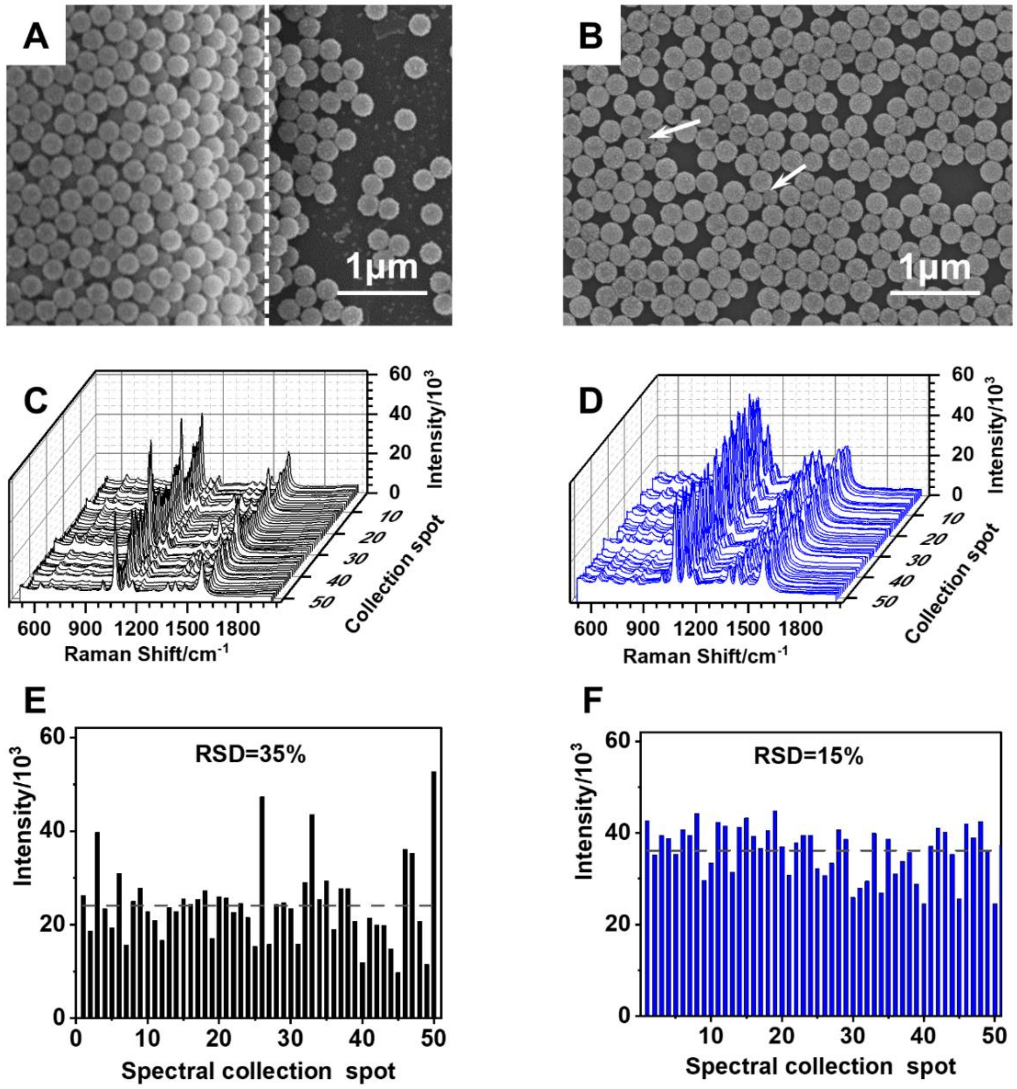

Figure S9. (**A** and **B**) SEM images of non-PC substrates from SiO_2@nAg with polydispersity index at (A) << 2% or (B) >2% used for (**C** and **D**) SERS of pATP to show (**E** and **F**) the variations of the peak intensity at 1087 cm^{-1} among 50 measurements, respectively.

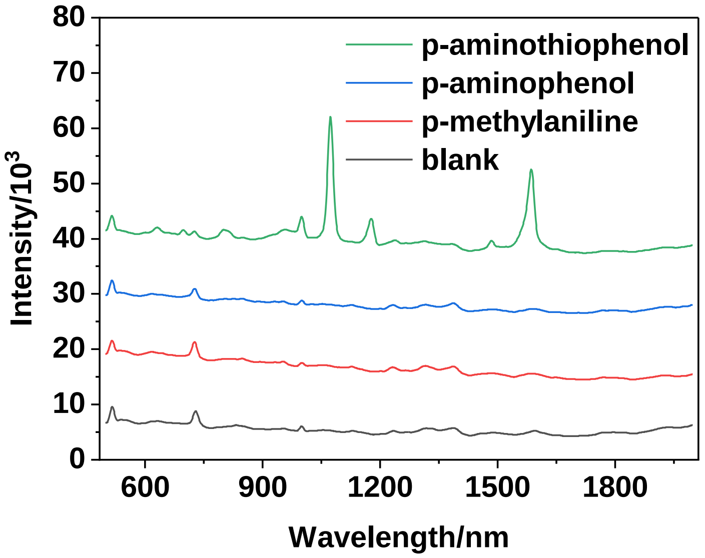

Figure S10. Raman spectra of different chemicals with or without thiol groups added in a diluted apple juice sample.

Evaluation of Enhancement Factor:

The enhancement factor (EF) was determined using the following equation:

$$EF=\frac{I_{SERS}\times N_{ref}}{I_{ref}\times N_{SERS}} \quad (1)$$

In our case, the experiments were carried out in similar condition using solution of pATP in presence and absence of PCs substrate. I_{SERS} and I_{ref} were found to be 42000 and 258 counts respectively (taking the peak intensity of 1078 cm^{-1} Raman peak).

N_{ref} was calculated by using the formula [1]:

$$N_{ref} = B_V \times D_{pATP} \times A/M_{pATP} \quad (2)$$

Where Bv is the optical excitation volume, D_{pATP} is the density of pATP (1.06 g/cm^3) in the reference bulk crystal, A is the Avogadro number, and M_{pATP} is the molar mass of pATP (125.19 g/mol). The excitation volume was estimated as the product of the laser spot (diameter of laser beam spot ca. 105 μm) and the depth of focus [2] $D_f \approx 2\lambda/(N.A.)^2$, where λ=785 nm is the excitation wavelength and N.A.=0.4 is the numerical aperture of the objective, thus yielding $D_f \approx$ 10 μm. N_{ref} was determined to be 4.41×10^{14}.

As a homogenous film was formed on monolayer PCs substrate, we can assume a homogeneous distribution of analyte. As 10 μL of 10^{-6} M (Total mole = 10^{-11} in 10 μL volume) solution in ethanol was spread onto the substrate over 4 mm × 4 mm area, the surface concentration (i.e. the surface absorbed analyte) was found to be 6.25×10^{-19} mol/μm^2. Here, diameter of laser beam spot is 105 μm. So, the number of molecules (N_{SERS}) excited by laser was found to be 3.26×10^9 (obtained by multiplying the surface concentration with laser spot area and Avogadro constant). Based on above data, EF was calculated to be ~ 2.2×10^7. The actual value of EF might be higher by an order as we assumed the maximum value of absorbed analyte concentration.

Evaluation of Limit of Detection:

Limit of detection (c_m) was calculated using the following expression:

$$c_m = \frac{S_m - S_{bl}}{S} = \frac{ks_{bl}}{S} \quad (3)$$

where S_m is the minimum distinguishable signal, S_{bl} is the Raman signal generated by a blank measurement of the SERS substrate in the absence of the analyte, S is calibration sensitivity, k is the proportionality constant, and s_{bl} is the standard deviation of blank measurements. To measure reliably, we carried out 35 blank measurements from 7 seven replicate substrates (S_{bl}=1133, s_{bl}=94.8). The reasonable value of k is k=3, and the detection confidence level is 95% in most cases. From the Figure 6 in the manuscript, we known S=8.19. So, c_m=3*94.8/8.19=34.7 ppb. This is a standardized in analytical chemistry.

Reference

1. Gopinath A; Boriskina S V; Reinhard B M.; Negro L D. Deterministic aperiodic arrays of metal nanoparticles for surface-enhanced Raman scattering (SERS)[J]. *Optics Express*. **2009**, *17(5)*, 3741–3753.
2. Alvarez-Puebla R A. Effects of the Excitation Wavelength on the SERS Spectrum[J]. *Journal of Physical Chemistry Letters*. **2012**, *3(7)*, 857–866.
